# Population attributable fractions of clinical and social risk factors for suicide in Bangladesh: Finding from a case–control psychological autopsy study

**DOI:** 10.1002/brb3.2409

**Published:** 2021-11-10

**Authors:** S. M. Yasir Arafat, Md. Abdullah Saeed Khan, Duleeka Knipe, Murad M. Khan

**Affiliations:** ^1^ Department of Psychiatry Enam Medical College and Hospital Dhaka Bangladesh; ^2^ Pi Research Consultancy Center Dhaka Bangladesh; ^3^ Population Health Sciences, Bristol Medical School University of Bristol Bristol UK; ^4^ Department of Psychiatry Aga Khan University Karachi Pakistan

**Keywords:** case–control study, population attributable fraction, psychological autopsy, risk factors, suicide in Bangladesh

## Abstract

**Background:**

Our knowledge of suicide in low‐income countries is limited. Understanding the importance of factors that contribute to suicide risk will allow for the appropriate allocation of limited resources. In order to prioritize suicide prevention activities in Bangladesh, we estimate the fractions of suicides attributable to key risk factors.

**Methods:**

Using data from matched cases (100) and controls (100) as part of a psychological autopsy study in Dhaka, we estimate the population attributable fraction for key clinical (psychiatric disorders and physical disability), and social (life events, psychical and/or sexual abuse, unemployment, and social isolation) risk factors for suicide in Bangladesh.

**Results:**

Assuming a causal relationship, life events were responsible for the largest proportion of suicide deaths (85.9%; confidence interval [CI], 79.6–90.2), followed by mental disorder (49.5%; CI, 45.3–53.4). The population attributable fraction for the risk factors was 42.9% (CI, 40.6–45) for depression, 11% (CI, 8.9–13) for sexual abuse, and 34.9% (CI, 10.1–52.9) for social isolation.

**Conclusions:**

The study determined the population attributable fraction of risk factors for suicide in Bangladesh. Prevention strategies should be prioritized on the management of the aftermaths of adverse life events, treatment of psychiatric disorders, sexual abuse, and social isolation in the country.

## Abstract 2

This is the first study determining the population attributable fraction of risk factors for suicide in Bangladesh. Adverse life events were responsible for the largest proportion of suicide, followed by mental disorders. Prevention strategies should focus on the management of key social and clinical risk factors.

## 1 INTRODUCTION

Suicide is a global public health problem. It is the outcome of complex interactions among biological, psychological, social, cultural, and religious factors (World Health Organization [WHO], 2014). Several models have been proposed to explain the complex interaction between the domains of risk factors for suicide such as the stress‐diathesis model, gene‐environment interaction, and proximal and distal risk factors (Li et al., 2011; WHO, 2014; Zalsman et al., 2016). Over the past decades, considerable progress has been observed in suicide research, while the prevention of suicide is yet to gather momentum (Krysinska & Martin, 2009). Identifying the risk factors for suicide is a fundamental component of any effective response in suicide prevention (WHO, 2014). However, precise estimation of risk factors is still nearly impossible (Bolton & Robinson, 2010; WHO, 2014; Zalsman et al., 2016). Several areas such as psychiatric disorders, adverse life events, previous nonfatal attempts, and social isolation have been identified as the risk factors by well‐designed psychological autopsy studies (Bolton & Robinson, 2010; Cavanagh et al., 2003). Nevertheless, there has been a dearth of studies estimating the population attributable fraction (PAF) to assess the impact of protective factors and the role of preventive measures in suicidology (Afifi et al., 2008; Bolton & Robinson, 2010; Krysinska & Martin, 2009; Park et al., 2016). The PAF allows for an estimation of the relative importance of risk factors and for the prioritization of suicide prevention efforts. It indicates the reduction in the incidence of a particular outcome in the population if the risk factor could be removed (Bolton & Robinson, 2010; Park et al., 2016).

Suicide and its prevention are understudied public health issues in Bangladesh where attempts to identifying the risk factors are inadequate (Arafat, 2019). The stigma associated with suicide and the criminal status of suicidal behavior hinders disclosure and help‐seeking. One recent systematic review identified only two case–control studies in the last decade including one psychological autopsy study and no longitudinal study (Arafat, Hussain, et al., 2021). The earlier case–control study was conducted in a rural setting and identified personal issues and emotional factors, previous nonfatal attempts, and familial disharmony as the major risk factors (Reza et al., 2013). Another systematic review identified proximally associated personal emotional issues, marital discord, and familial disharmony as the major risk factors for suicide (Arafat, 2019). Psychiatric disorders have been poorly studied as risk factors for suicide in the country. The lone matched case–control psychological autopsy study identified the presence of an adverse life event, mental illness, past nonfatal attempt, sexual violence, unemployment, physical abuse, and social isolation as risk factors for suicide in the country (Arafat, Mohit, et al., 2021). Nevertheless, the impact of any individual risk factor for suicide and the impact of preventing of that factor on the general population have not been determined. No prevention strategy has been identified based on these risk factors. Studies assessing the population attribution risk (PAR) and PAF, which is an extremely important public health aspect of suicide prevention, could not be identified in the country. Henceforth, we aimed to estimate the PAF for key clinical (psychiatric disorders and physical disability) and social (life events, psychical/sexual abuse, unemployment, and social isolation) risk factors in the general population of Bangladesh. The findings would be necessary to prioritize suicide prevention activities in Bangladesh while formulating the national suicide prevention strategy.

## 2 METHODS

### 2.1 Study setting

Bangladesh, a South Asian lower middle‐income country, is densely populated with a population of more than 160 million people. It is a Muslim majority country where suicide has been still criminalized (World Population Review, 2021a; United for Global Mental Health, 2021). The 2019 estimates revealed the age‐standardized suicide rate in Bangladesh was 6, 1.7, and 3.9 per 100,000 population among males, females, and both sexes, respectively, which is lower when compared to the global (9 per 100,000) and regional (South East Asia: 10.2 per 100,000) rates (WHO, 2021). However, under‐reporting of suicides hides the actual rate, and the credibility of suicide data has been challenged in the country. We conducted the study in Dhaka city, the capital of Bangladesh, which is also one of the rapidly expanding megacities in the world. Dhaka has more than 20 million people with a density of about 23,234 people per square kilometer (World Population Review, 2021b). We collected the list of suicides from the forensic medicine department of two medical colleges of Dhaka city and visited the next of kins’ home for an interview.

### 2.2 Data collection

We analyzed the data of a case–control psychological autopsy study conducted between July 2019 and July 2020. The dataset includes semistructured interview responses of 100 suicide deaths and 100 living controls matched for age, gender, and living area those were collected in the Dhaka Suicide Study project (Arafat, Mohit, et al., 2021). Structured Clinical Interview for DSM‐IV Axis‐I Disorders (SCID‐I) (First et al., 1996) and the Structured Clinical Interview for DSM‐IV Personality Disorders (SCID‐II) were used to determine mental disorders (First et al., 1994). We identified life events from the list of Paykel's Life Events Schedule (Paykel et al., 1971). Additionally, we noted some culturally important life events that were not mentioned in the schedule (e.g., sexual harassment) (Arafat, Mohit, et al., 2021). The next of kins of suicides and controls were interviewed by the first author (psychiatrist) by face‐to‐face interview technique. More detail of methods has been mentioned in our previous published paper of the matched case–control psychological autopsy study (Arafat, Mohit, et al., 2021).

### 2.3 Data analysis

Sociodemographic profile, substance abuse, physical abuse, sexual harassment, life events, and psychiatric disorders of case and controls were explored for the potential determinants of suicide. Univariate conditional logistic regression models were run to extract the matched odds ratio (ORs) and their 95% confidence intervals (CIs) (Arafat, Mohit, et al., 2021). In this study, the regression was performed in STATA version 16.0 using the *clogit* function. Major depressive disorder, physical disability, physical abuse, sexual abuse, life events, employment status, and social networks were considered in the univariate analysis as these factors revealed statistical evidence of association. PAFs (%) were calculated using *punafcc* postestimation function in STATA based on the method described by Greenland and Drescher (1993).

### 2.4 Ethical approval

Ethical approval of the Dhaka suicide study was taken from the ethical committee of the National Institute of Mental Health, Dhaka (NIMH/2019/1053). Informed written consent was taken from the respondents.

## 3 RESULTS

### 3.1 Baseline characteristics

The mean age of the cases and controls were 26.30 (±12.36) and 26.68 (±11.96) years, respectively. Among the 100 suicides, 51 were females, and 72 were living in the periphery of the city. There was no statistical evidence of a difference between cases and controls based on marital status and religion. Detailed demographic characteristics have been discussed in our initial paper of the dataset (Arafat, Mohit, et al., 2021). Among the cases, 61% had at least one psychiatric (Axis I and/or Axis II) disorder, while 13% of the controls had the same (Table 1). Among the suicides, 44% had depression, while 6% of the controls had the same (Table 1). For cases, 91% had life events during the last year in comparison to the 24% of the controls (Table 1).

**TABLE 1:** Population attributable fraction of key risk factors for suicide in Bangladesh

Risk factor	Cases** (%)	Controls** (%)	OR* (95%CI)	PAF% (95%CI)
Life events				
Absent	9	76	1	
Present	91	24	17.75 (6.48–48.59)	85.87 (79.64–90.20)
Social networks				
Many friends	45	28	1	
Isolated or few friends	72	55	1.94 (1.01–3.43)	34.97 (10.12–52.95)
Sexual abuse				
No and others	88	99	1	
Yes	12	1	12 (1.56–92.29)	11 (8.94–13.02)
Physical abuse				
No and others	87	95	1	
Yes	13	5	3 (0.96–9.30)	8.67 (3.63–13.44)
Employment status				
Employed and others	84	95	1	
Unemployed	16	5	4.67 (1.34–16.24)	12.57 (8.18–16.74)
Psychiatric disorder				
Absent	39	87	1	
Present	61	13	15.33 (4.76–49.30)	49.54 (45.34–53.42)
Axis I disorder				
Absent	43	91	1	
Present	57	11	16.33 (5.09–52.40)	53.51 (49.26–57.41)
Major depressive disorder (MDD)				
Absent	56	94	1	
Present	44	6	39 (5.36–283.86)	42.87 (40.59–45.07)
Substance abuse				
Absent	91	99	1	
Present	9	1	9 (1.14–71.04)	8 (5.91–10.04)
Personality disorder				
Absent	86	98	1	
Present	14	2	13 (1.70–99.37)	13.05 (10.81–15.24)
Physical disability				
Absent and others	96	99	1	
Present	4	1	4 (0.45–35.79)	3 (0.78–5.17)

Abbreviations: CI, confidence interval; OR, odds ratio; PAF, population attributable fraction.

* Determined by univariate conditional logistic regression analysis.

** *n* = 100.

### 3.2 Population attributable fraction

The PAFs for the risk factors that showed statistical evidence of an association are presented in Table 1. Adverse life events were the highest (85.9%) attributing fraction, followed by the presence of a mental disorder (49.5%), social isolation (about 35%), unemployment 12.6%, and sexual violence (11%) (Table 1). The PAF was 53.5% (49.26–57.41) for Axis I disorders, 42.9% (CI, 40.6–45) for depression, 8% (CI, 5.9–10) for substance abuse, and 13% (10.81–15.24) for personality disorders (Table 1).

## 4 DISCUSSION

### 4.1 Main findings of the study

The current study revealed that social risk factors (life events, psychical and/or sexual abuse, employment, and social isolation) were predominantly attributable risk factors for suicide in Bangladesh (Table 1). Among the social risk factors, adverse life events shared the most significant fraction and had a link with about 86% of suicides in the general population. A detailed list of the life events has been mentioned in our initial paper (Arafat, Mohit, et al., 2021). Other social factors indicated that social isolation attributed to about 35% of suicides, followed by unemployment that attributed to about 13%, sexual abuse (11%), and physical abuse about 9% (Table 1). The clinical risk factors (any psychiatric disorder and physical disability) were identified as the second most important domain determined by the PAF. The presence of a psychiatric illness attributed to about 50% of suicides, followed by the presence of at least one Axis I disorder mental illness (about 53.4%), depressive disorder (about 43%), substance abuse (8%), and personality disorder attributed to 13% of the suicides in the general population. Although the past suicidal attempt was found as an important risk factor in our primary paper, we dropped it from the analysis of PAF because the univariate conditional regression model in STATA revealed an implausible value (Arafat, Mohit, et al., 2021). Previously, we analyzed the data by SPSS and JASP while extracting the ORs by univariate conditional logistic regression (Arafat, Mohit, et al., 2021).

### 4.2 Implications

There has been a dearth of studies identifying the PAF in suicidology that has an enormous impact on policymaking, designing the preventive strategies, and assessing the effectiveness of suicide prevention interventions (Krysinska & Martin, 2009; Park et al., 2016). Therefore, we estimated the fractions of suicides attributable to clinical and social risk factors in the general population of Bangladesh from our matched case–control psychological autopsy data. Prevention strategies should be prioritized based on the identified areas from the PAFs. For example, prevention strategies for coping with adverse life events could reduce about 86% of suicides in the general population of Bangladesh. Similarly, adequate mental health services to treat the Axis I disorder and depression would reduce 53.5% and about 43% of suicides, respectively. There are undeniable and complex interactions between the key domains of clinical (psychiatric disorder and physical disability) and social risk factors (adverse life events, social isolation, physical and/or sexual abuse, and unemployment). Previous studies also revealed the interactions between the domains of risk factors at least between social risk factors and psychiatric disorders (Li et al., 2011).

Our result is consistent with previous studies assessing the PAFs of risk factors for suicide as per the domains. However, there are significant differences between the proportions of risk factors in different studies. A systematic review of psychological autopsy studies identified the PAF for psychiatric disorders ranged from 47% to 74% (Cavanagh et al., 2003). Another systematic review found that the PAF for mood disorders was 11% (Li et al., 2011). One national representative study conducted in the United States revealed PAF was 36.6% for mood disorders, 31.8% for any substance use disorder, 4.9% for psychotic disorders, and 30.2% for personality disorders (Bolton & Robinson, 2010). Another study in Korea revealed that depression was attributable for 45.7% of suicidal ideation among the elderly persons, followed by chronic diseases (19.4%), poor subjective health status (18.9%), sleep disturbances (14.1%), functional impairment (4.9%), low social support (4.2%), and active smoking (3.6%) (Park et al., 2016). Studies assessing the adverse childhood experience revealed that physical and sexual abuse have been associated with suicide (Afifi et al., 2008; McLafferty et al., 2018). Among the available studies, there is the heterogeneity of methods and PAFs; however, there is gross homogeneity in the domains of risk factors (Afifi et al., 2008; Cavanagh et al., 2003; Li et al., 2011; McLafferty et al., 2018). It is important to note that these studies have been conducted in high‐income country settings, while our study represents a low‐ and middle‐income country setting. It is prudent to note that the current study revealed social risk factors as the leading area to consider the prevention strategies based on the PAF, followed by the clinical risk factors (Table 1). Recent reviews identified a possibility of a lower prevalence of mental disorders in suicidal behavior in low‐ and middle‐income country settings (Knipe et al., 2019), and in WHO South East Asia region (Arafat, Menon, et al., 2021). Additionally, existing stigma toward suicide and mental disorders and poor mental health literacy could hinder the identification of psychiatric disorders among the suicides in Bangladesh (Arafat, Mohit, et al., 2021).

Adequate psychiatric services to treat psychiatric disorders have no alternative as a prevention strategy in Bangladesh. Awareness and mental health literacy are the necessities to identify depression and substance abuse by the community gatekeepers, and family members. General physicians should be adequately trained to diagnose and treat the disorders (Arafat & Kabir, 2017; Zalsman et al., 2016). Universal prevention strategies such as raising awareness among family members regarding the consequences of adverse life events, coping with distress, and dedicated helplines should be considered to prevent suicides related to adverse life events (Arafat & Kabir, 2017; Arafat, Mohit, et al., 2021; Zalsman et al., 2016). The family members should be well oriented regarding the risk of fatal attempts after a life event since marital discord and familial disharmony are potential identified life events (Arafat, Mohit, et al., 2021). Also, special attention should be paid to sexual harassment as a risk factor of suicide in Bangladesh. Any recent loss of a job or unemployment for a longer duration is an important area to address. Long‐term strategies should be considered for socially isolated individuals. Decriminalization of suicide could reduce the stigma and increase the help seeking for self‐harm and suicide in the country (United for Global Mental Health, 2021).

### 4.3 Strengths and limitations

This is the first study determining the PAF of risk factors for suicide in Bangladesh which would guide to prioritize the prevention strategies in Bangladesh. However, a prudential interpretation is warranted due to several potential limitations. First, the study was conducted in the capital city of the country which is an urban setting and may not represent the population of the whole country. The distribution of life events and psychiatric disorders could vary when it is compared to the rural setting of the country. Second, the response rate was low both in cases and controls which is a potential area of biases and warrants the generalization of study results. Third, interviews were conducted purposively, which also challenges the generalization of study results. Masking was not done while interviewing the next of kins of cases and controls that may be a source of interview bias. Fourth, we were unable to adequately estimate the association between past suicide attempts and suicide in our analysis and therefore estimate PAF because there were too few cases and controls with this outcome in our matched study. Fifth, there might have chances of recall biases during the interview as it is more likely to recall factors in cases than controls. Therefore, under‐reporting of life events and psychiatric symptoms is more likely in the control group. Finally, PAF reveals the causal relationship between the exposure and the outcome which may not completely fit in suicidology because suicide happens as a result of a complex interaction among multiple risk factors. Current evidence does not support any mono‐causal explanation of suicide. Therefore, an extreme caution is warranted while asserting the causal role with any individual factor and suicide.

## 5 CONCLUSION

The study determined the PAF of key social and clinical risk factors for suicide in Bangladesh. Prevention strategies should focus on the management of adverse life events, treatment of psychiatric disorders, sexual abuse, unemployment, and social isolation.

## CONFLICT OF INTEREST

The authors declare no conflict of interest.

## AUTHOR CONTRIBUTIONS

*Conception & Design*: Murad M. Khan. *Acquisition of data*: S. M. Yasir Arafat. *Data analysis plan*: Md. Abdullah Saeed Khan and Duleeka Knipe. *Data analysis*: Md. Abdullah Saeed Khan. *Drafting of the manuscript*: S. M. Yasir Arafat. *Critical revision and final approval of the manuscript*: All authors.

### PEER REVIEW

The peer review history for this article is available at https://publons.com/publon/10.1002/brb3.2409

## Data Availability

All data are provided in this study and raw data can be requested from corresponding author.
